# From Multimodal Sensorimotor Integration to Semantic Networks: A Phylogenetic Perspective on Speech and Language Evolution

**DOI:** 10.1162/nol_a_00164

**Published:** 2025-04-24

**Authors:** Maëva Michon, Francisco Aboitiz

**Affiliations:** Praxiling Laboratory, UMR 5267, CNRS, Université Paul Valéry, Montpellier, France; Laboratory for Cognitive and Evolutionary Neuroscience, Interdisciplinary Center for Neuroscience, Department of Psychiatry, Faculty of Medicine, Pontificia Universidad Católica de Chile, Santiago, Chile

**Keywords:** language evolution, multimodal integration, perisylvian regions, semantic network, sensorimotor, superior temporal sulcus (STS)

## Abstract

This integrative perspective article delves into the crucial role of the superior temporal sulcus (STS) and adjacent perisylvian regions in multimodal integration and semantic cognition. Drawing from a wide range of neuroscientific evidence, including studies on nonhuman primates and human brain evolution, the article highlights the significance of the STS in linking auditory and visual modalities, particularly in the establishment of associative links between auditory inputs and visual stimuli. Furthermore, it explores the expansion of the human temporal lobe and its implications for the amplification of multisensory regions, emphasizing the role of these regions in the development of word-related concepts and semantic networks. We propose a posteroanterior gradient organization in the human temporal lobe, from low-level sensorimotor integration in posterior regions to higher-order, transmodal semantic control in anterior portions, particularly in the anterior temporal lobe. Overall, this perspective provides a comprehensive overview of the functional and evolutionary aspects of the STS and adjacent regions in multimodal integration and semantic cognition, offering valuable insights for future research in this field.

## INTRODUCTION

Traditionally, the research on sensory neuroscience has long been divided along modality-specific lines, with experts in the field of auditory or visual or motor systems. This dominant approach has led to a focus on questions concerning specific primary sensory pathways. Not until more recently did a field of research on multisensory neuroscience emerge and consolidate (Stein et al., 2020). In the past, our understanding of how we perceive objects was primarily grounded in visual processing alone. The neuroscience of vision revealed that to achieve object identification and localization, different types of information are processed in different neural pathways. Audition neuroscientists have then shown that objects can also be perceived auditorily with dedicated brain pathways to localize and identify sounds. It seems evident that, additionally to shape and sound (i.e., visual and auditory information) different features, like weight, texture, and even taste or smell, can be used to perceive individual objects, not just their shape. These multisensory representations probably enhance object perception, making it more reliable (Newell et al., 2023). Importantly, at the crossroads between seemingly sensory-specific pathways, subcortical and cortical areas with multisensory response profiles have been described. For instance, neurons in the superior colliculus and temporal sulcus display responses to multisensory stimuli that exceed the linear sum of individual responses to sensory-specific stimuli presented separately. The discovery of such a property, known as super-additivity, has had critical implications for the neuroscience of multisensory integration (Stein & Meredith, 1993).

The capacity of neurons to integrate multisensory information is not innate. Instead, it develops with repeated exposure to pieces of sensory information perceived concomitantly and accumulating substantial cross-modal experience (Bean et al., 2023; Smyre et al., 2024). Our interactions with the world are multisensorial and our conceptual knowledge about it requires cross-modal (visual, auditory, tactile, gustatory, somatosensory, and motor) integration (Lambon Ralph, 2014), resulting in a meaningful, adaptative, and coherent perception of the world (Gomez-Marin & Ghazanfar, 2019; Varela et al., 2017). Recent research has suggested that the representation of concepts is largely rooted in our experiential knowledge, involving both action and perception (Fernandino et al., 2016; Kuhnke, Kiefer, & Hartwigsen, 2023; Pouw et al., 2021; Tong et al., 2022). Congruently, there is increasing support for the idea that sensorimotor representations upon which concepts were acquired are reactivated for the comprehension of words conveying concepts (Fernandino & Conant, 2023; Fernandino et al., 2022; Kuhnke et al., 2020). Based on statistical associative learning (i.e., the detection of statistical regularities such as co-occurrences between stimuli in our environment), the human brain is able to generate multisensory predictions. For example, young children can associate the word “dog” with the shape of a dog and the barking of a dog.

This perspective article aims to provide a comprehensive overview of the functional and evolutionary aspects of the superior temporal sulcus (STS) and perisylvian regions in multimodal integration and semantic cognition. In the next section, we highlight the role of sensory and motor systems in conceptual knowledge, suggesting that modality-specific sensorimotor brain regions and multimodal convergence zones are engaged to give meaning to the world. A Multimodal Interface Along STS addresses the similarities in organizational structures and processing pathways between the auditory and visual systems, emphasizing the integration of multimodal sensory information in the STS and temporoparietal regions. This multimodal interface is crucial for understanding how the brain processes and integrates sensory information within the context of social interaction and communication. In Specialization of the pSTS During Brain Evolution, we examine the possible mechanisms by which this multimodal interface in the temporal lobe has consolidated during brain evolution, allowing the emergence of a proto-language in our ancestors. We and others have proposed that the expansion of the cortical regions associated with the STS facilitated the generation of multimodal representations including sound-object associations, amplifying multimodal representations that provided an early scaffolding for the emergence of semantic links to proto-linguistic utterances, that is, names for objects (Aboitiz, 2018a, 2018b; Kausel et al., 2024; Petrides, 2023). Finally, we discuss the anatomo-functional and phylogenetic constraints of the Homo lineage which may have enabled our ancestors to develop an increasing vocal repertoire, leading to the development of early human language.

### From Representations to Reenactment: The Re-Engagement of Multimodal Experiences

During oral communication, understanding the meaning of a word is thought to imply an association between perceived speech sounds forming words and the physical aspects of the referred object, person, or action afforded by our sensory systems. Objects, people, or action recognition can be performed via different sensory modalities. A person can be recognized by her face or voice because we have learned to associate the identity of this person, the sound of her name with the sound of her voice and how she looks (audiovisual associations). Fruits can be recognized by associating their names with their shapes, colors, tastes and smells (Lambon Ralph, 2014). Across languages, words (particularly nouns and action verbs) are related to different modalities of sensory experience, and their representations are multimodal (Ibáñez et al., 2023; Lynott et al., 2020; Miklashevsky, 2018; Speed & Brysbaert, 2024; Speed & Majid, 2017; Vergallito et al., 2020; Zhong et al., 2022). A recent neuroimaging study has evidenced a distributed network for multimodal experiential representation of concepts and showed that “the retrieval of conceptual knowledge during word comprehension relies on a much larger portion of the cerebral cortex than previously thought and that multimodal experiential information is represented throughout the entire network” (Tong et al., 2022, p. 7121). This implies that linguistic concepts rely on multimodal, possibly associative networks distributed across different cortical regions (Lambon Ralph et al., 2017).

Rather than understanding meaning as the processing of amodal, encapsulated representations, an increasing number of scientists is advocating for the grounding of meaning in the reenactment of these multimodal experiences with the world (Borghi et al., 2024; Calzavarini, 2024; Dove, 2023a; Kewenig et al., 2024; Pulvermüller, 2013). This approach, in our opinion, is biologically more plausible because living organisms learn about and understand the world by purposefully interacting with their environment and predicting the outcomes of those interactions. This process helps them to develop a fundamental understanding and a sense of significance, which becomes the ground for their subsequent knowledge (Pezzulo et al., 2024). From this standpoint, the living organism is not a passive observer of an external, predetermined reality but rather a proactive agent that constructs its own reality by interacting with its environment and predicting the sensory consequences of its action. A notion compatible with this view, called *active inference*, proposes that sensory and motor systems are actively recruited during perception (Parr et al., 2022; Ramstead et al., 2020; Schroeder et al., 2010). Through repeated interactions with its environment, an organism can identify regularities and extract sensorimotor contingencies. Importantly, the organism interplays with the environment through its actions and eventually becomes able to predict the changes its own or others’ actions can produce in the somatosensory input. According to this framework, the semantic content of a concept is achieved by reusing the perceptual and motor areas of the brain that allow us to sense and interact with the world (Dove, 2023b, 2024; Pulvermüller, 2013; Pulvermüller & Fadiga, 2010).

Importantly, the grounding of conceptual knowledge does not seem to be limited to concrete concepts. Using functional magnetic resonance imaging (fMRI), Harpaintner and collaborators (2020) found that processing abstract concepts with a known motor (e.g., effort) versus visual (e.g., beauty) feature content respectively activates action-related and vision-related brain regions, as identified in single subjects’ brains by functional localizer tasks (hand movement for the motor localizer and picture exploration for the visual localizer). A recent meta-analysis of 212 neuroimaging studies reported that “conceptual processing consistently engages brain regions also activated during real perceptual-motor experience of the same modalities” (Kuhnke, Beaupain, et al., 2023, abstract). The authors propose a novel hierarchical model for conceptual knowledge engaging not only modality-specific sensorimotor brain regions but also multimodal convergence zones, such as the inferior parietal lobe and the posterior middle temporal gyrus. In the remaining sections of this perspective article, we address the nature and functional relevance of these multimodal convergence zones for speech and language with greater detail.

### A Multimodal Interface Along STS

In their inaugural model, Mortimer Mishkin and Leslie Ungerleider (1982) initially outlined a dual pathway arrangement of the visual system in the nonhuman primate brain. This organization featured a ventral stream specialized in object recognition and a dorsal stream dedicated to determining objects’ spatial location. Ten years later, a comparable arrangement was described in the human visual system (Goodale & Milner, 1992). This anatomo-functional organization was then expanded to the auditory system of both nonhuman (Kaas & Hackett, 1999; Rauschecker & Tian, 2000) and human primates (Arnott et al., 2004), highlighting a ventral “what” and a dorsal “where” stream specialized for sound recognition and spatial localization, respectively. The description of this functional organization in “low-level” sensory cortices has subsequently inspired dual-pathway theories for “higher-order” cognitive functions, such as attention, exemplified by the ventral and dorsal attentional network proposed by Corbetta and Shulman (2002), and language, illustrated by the ventral and dorsal streams for language posited by Hickok and Poeppel (2007). Nearly 40 years after Mishkin and Ungerleider’s (1982) original proposition, however, there is growing evidence indicating the need for an update to the dual-stream model of the visual pathway.

### The Visual System

**Figure 1.**
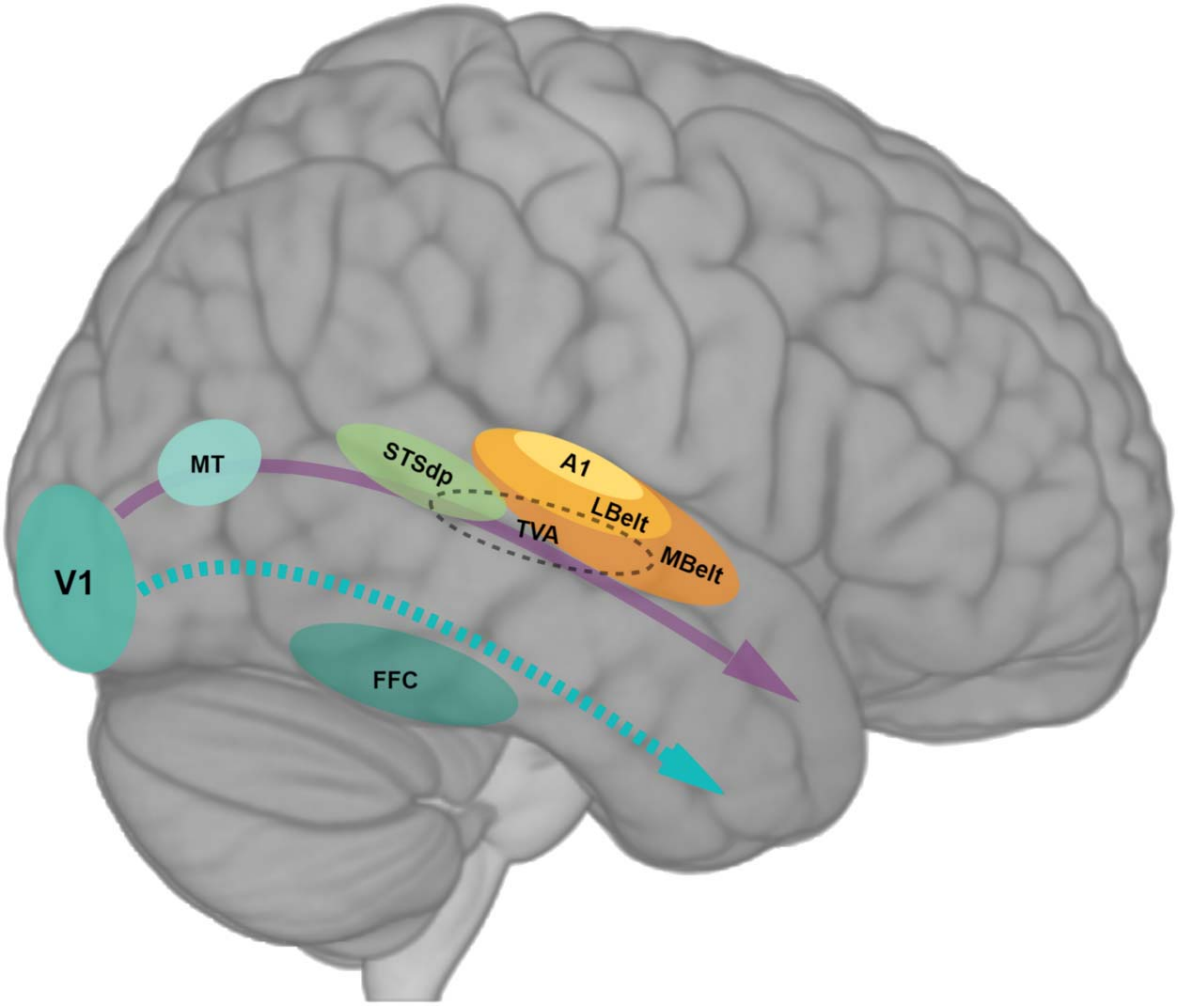
Right STSdp as an interface for multimodal integration of social stimuli. Sensory-specific systems process visual and auditory social stimuli such as faces and voices in the occipital and temporal gyri. Along the lateral surface of the brain, at the interface of auditory (yellow and orange areas) and visual (blue areas) regions, stands STSdp (green area), which is increasingly accepted as an area supporting multisensory integration. The lateral visual stream (solid purple arrow) is anatomically and functionally different from the ventral visual stream (dashed blue arrow), the former dealing with dynamic, animated aspects of social perception and action recognition while the latter seems to respond to static or unanimated objects and people. (Areas depicted by solid colored circles are named after the Human Connectome Project nomenclature). V1 = primary visual cortex; FFC = fusiform face cortex; MT = middle temporal area; A1 = primary auditory cortex; Lbelt = lateral auditory belt; MBelt = medial auditory belt; TVA = temporal voice area; STSdp = superior temporal sulcus dorsal posterior.

It was recently proposed that the dual stream model for the organization of the visual system should be updated to include a third, lateral pathway specialized for dynamic social perception (Pitcher, 2021; Pitcher & Ungerleider, 2021). The authors argue for a third stream anatomically and functionally independent from the ventral stream (see purple and blue arrows in Figure 1), which projects on the lateral surface of both humans’ and macaques’ brains, from primary visual cortex (V1) to anterior STS (aSTS), encompassing visual motion visual middle temporal area (V5/MT), extrastriate body area (EBA) and the posterior STS (pSTS). This third visual stream is thought to be involved during the processing of socially relevant dynamic biological motion such as body posture and facial expression and to support, along with other regions of the temporoparietal union, higher-order functions like social cognition and theory of mind. Recently, using transcranial magnetic stimulation, Gandolfo et al. (2024) reported the causal involvement of the left EBA in detecting visual signals of social interaction. Wurm and Caramazza (2022), on the other hand, have argued for a subdivision of the ventral “what” pathway into two streams; a ventral and a lateral stream specialized for object and action recognition, respectively. Interestingly, and in line with the proposal of a third visual stream “specialized for interpreting the physical actions that we use to understand others” (Pitcher, 2023, p. R1222), Wurm and Caramazza (2022) described a dorsoventral gradient in the organization of the lateral occipitotemporal cortex, with dorsal portions responding to animated entities and ventral portions to unanimated entities. They also propose a second dimension of organization of this lateral action stream along a posterior–anterior gradient from visual perceptual precursors to more specific semantic representations of dynamic actions and objects. Similar organizational principles of the visual system were formerly described by Weiner and Grill-Spector (2013), who proposed “a new model of high-level visual cortex consisting of ventral, lateral, and dorsal components, where multimodal processing related to vision, action, haptics, and language converges in the lateral pathway” (p. 74) with potential implications for social communication. More recently, McMahon and collaborators further explored the involvement and relevance of this lateral visual pathway for social interactions. Congruently with other proposals addressed above, they claim that the lateral visual pathway for social action recognition is hierarchically organized from posterior to anterior regions, with high-level social interaction information being processed along the STS (McMahon et al., 2023; McMahon & Isik, 2023, 2024).

### The Auditory System

**Figure 2.**
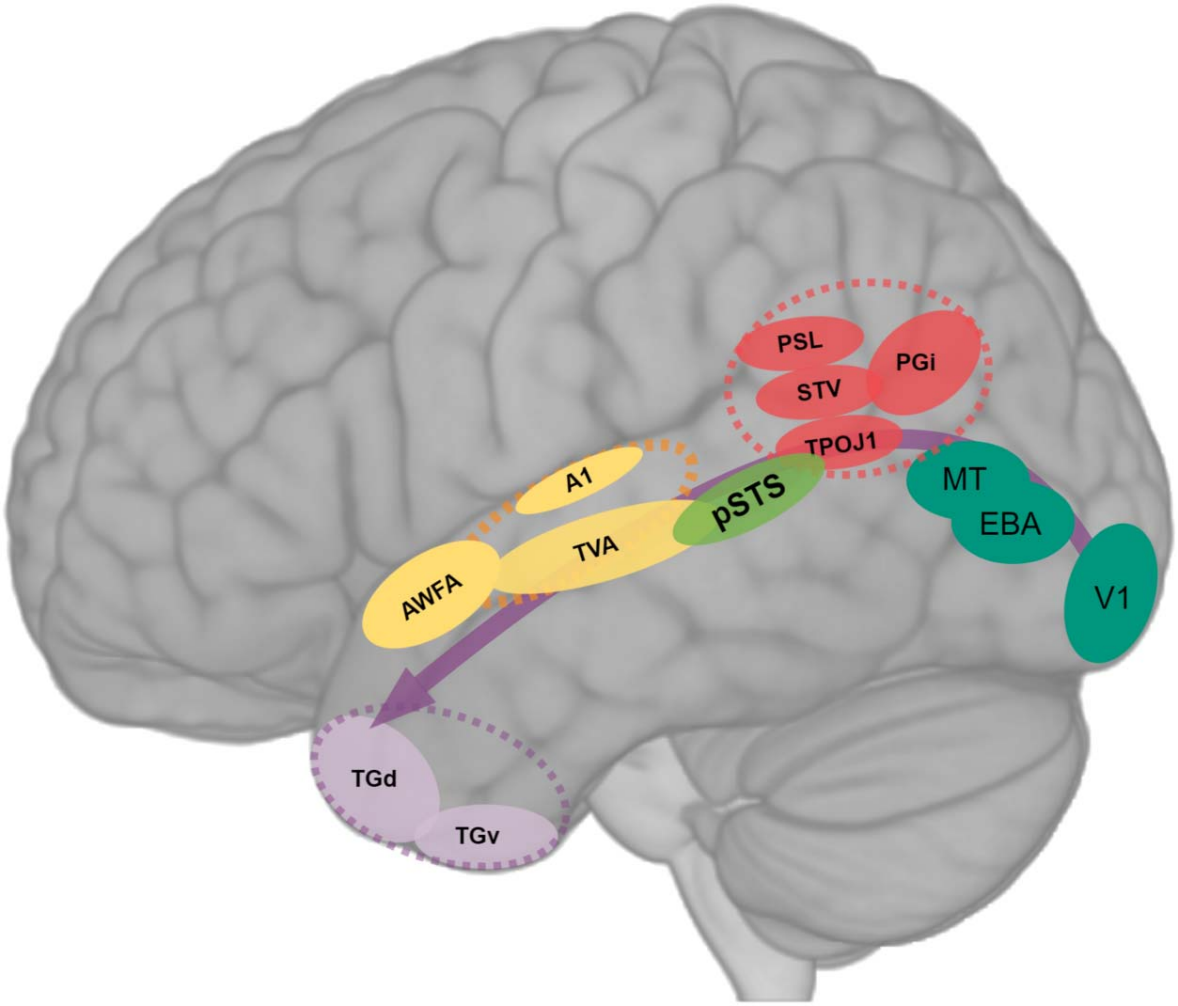
Evolution of a multimodal network for semantics. This neural circuit evolved from ancestral networks sustaining sensorimotor associations between modality-specific information to an expanded network enabling the mapping of linguistic labels (phonological and articulatory features of words and names) with multimodal information concerning the referred object, action or person. This network extends along the superior temporal sulcus and its progressive expansion in the left hemisphere allowed the consolidation of a proto-lexicon and the corresponding semantic relations with the empiric world. Dashed red circle indicates temporoparietal junction; dashed yellow circle = lateral auditory belt and medial auditory belt; dashed purple circle = anterior temporal lobe. V1 = primary visual cortex; MT = middle temporal area; EBA = extrastriate body area; PSL = perisylvian language area; STV = superior temporal visual area; PGi = inferior parietal PG area; TPOJ1 = temporoparietooccipital junction 1; pSTS = posterior superior temporal sulcus; A1 = primary auditory cortex; TVA = temporal voice area; AWFA = auditory word form area; TGd = lateral temporal TG dorsal; TGv = lateral temporal TG ventral.

Spoken words are produced by a specific sequence of movements executed by the speaker’s orofacial articulators (aka *articulemes*, Michon et al., 2019, 2020), which, once uttered, is perceived as a voice conveying a short stream of speech sounds in the listener’s brain. Extending bilaterally from the pSTS to the middle and anterior superior temporal gyri (mSTG and aSTG) stands an area known as the temporal voice area (TVA; depicted as a dashed ellipse in Figure 1). Compared to the auditory belts (depicted as yellow and orange ellipses in Figure 1), which are dedicated to the processing of general sound features, the TVA shows specific responsiveness to conspecific vocalizations over nonvocal sounds in the human brain (Agus et al., 2017; Belin et al., 2000; Bodin et al., 2021; Pernet et al., 2015). Single electrode recordings of neural activity in the human STG have revealed response selectivity to phonetic and acoustic features of continuous speech (Mesgarani et al., 2014). In non-human primates’ brains, neurons that respond selectively to species-specific vocalizations were previously identified in the anterior (or rostral) auditory belts of rhesus macaques (Rauschecker et al., 1995). Recently, similar regions for vocalization processing have been described in marmosets’ temporo-frontal cortices, suggesting that the voice perception circuit in the human brain may have evolved from a precursor circuit for vocalization processing predating the separation of the Old and New World primates (Belin et al., 2023; Jafari et al., 2023). It is noteworthy that the TVA at least partially overlaps with anterior auditory belt areas (depicted in dashed yellow circle in Figure 2), which were meticulously mapped with single-unit techniques in rhesus monkeys by Tian and collaborators (2001), and defined as functionally specialized for species-specific communication calls. More anteriorly, in the human ventral auditory stream, an auditory word form area was discovered in the left aSTG and called after its function for eliciting highly selective activity in response to spoken words, as opposed to written words which are processed in an area of the ventral visual stream called the visual word form area in the left fusiform gyrus (DeWitt & Rauschecker, 2012; Rolls et al., 2023). Reminiscent of the organizational principle of the visual ventral and lateral pathways, the ventral auditory stream seems to process increasingly complex vocal sounds (from phonemes to words to phrases) when moving forward to the anterior regions of the superior temporal lobe. Accordingly, new evidence suggests convergent functional organization strategies are present across modalities in the auditory and visual ventral streams (Damera et al., 2023).

### Multimodal Integration at the Crossroads of Visual and Auditory Systems

While distinct in their primary functions, the auditory and visual systems exhibit remarkable similarities in their organizational structures and processing pathways. Both systems are organized along a posteroanterior gradient, beginning with low-level perceptual features and advancing to anterior regions responsible for more complex semantic representations, such as speech sound processing in the auditory stream (Leaver & Rauschecker, 2010; Norman-Haignere et al., 2015) and object and action recognition in the visual stream (Wurm & Caramazza, 2022). This parallel organization highlights a convergent evolutionary strategy in the brain’s handling of different sensory modalities. Furthermore, recent research suggests that these sensory systems are not entirely isolated; they interact and integrate information, particularly in regions like the superior temporal lobe, which serves as a crucial interface. This interconnectedness is pivotal for understanding how the brain processes and integrates multimodal sensory information, setting the stage for examining the detailed pathways and their specialized functions within the context of social interaction and communication.

During social interactions, the faces and voices of interlocutors are often perceived together, simultaneously providing congruent visual and auditory communicative cues. In addition to the fusiform face and the occipital face areas in the ventral visual stream, STS dorsal posterior (STSdp) is also considered a classical core region for face processing. According to the evidence reported above in The Visual System, STSdp is involved particularly in response to dynamic as compared to static face processing, such as facial expressions but also visual speech perception. It is noteworthy that the STSdp receives inputs from both the ventral auditory stream and the lateral visual stream and is considered a hub for multimodal integration (Landsiedel & Koldewyn, 2023; Rolls et al., 2023). In nonhuman primates studies using single-unit recordings of brain activity, audiovisual integration has been reported in macaque face patch anterior fundus in STS, but not anterior medial in the anterior ventral temporal cortex (Ghazanfar et al., 2005; Khandhadia et al., 2021; Perrodin et al., 2014). Using fMRI in humans, Zhu and Beauchamp (2017) reported that the face-sensitive pSTS contains different neuronal populations that respond preferentially to eye movements or mouth movements and showed that only the subregion preferring mouth movements is also strongly responsive to voices (also see Rennig & Beauchamp, 2018). Congruent with the functional properties of this pSTS subregion, an area that is selective for visual speech versus nonspeech mouth movements has been identified in the left hemisphere. According to its function and location at the boundary of pMTG and pSTS, this area has been labeled the temporal visual speech area (TVSA; Bernstein et al., 2011; Bernstein & Liebenthal, 2014). Recently, using inhibitory transcranial magnetic stimulation, Jeschke et al. (2023) demonstrated the causal influences of area V5/MT, adjacent to the TVSA, on visual speech recognition. Importantly, as Rauschecker and Afsahi (2023) outlined, “STS regions also receive visual inputs about moving faces and objects, and the auditory and visual streams are combined to help in multimodal object identification, such as who is speaking, what is being said, what the object is, and so on” (Rauschecker & Afsahi, 2023, p. 1888). This connectivity pattern suggests that beyond its involvement in multimodal speech perception (Kausel et al., 2024), STS may also be crucial for grounded semantic cognition.

### Specialization of the pSTS During Brain Evolution

#### From action recognition to social cognition

Dorsal to area pSTS, the temporoparietal junction (TPJ) is largely accepted to be a key region of the social cognition network in both humans and nonhuman primates. Often considered as an anatomically ill-defined region, TPJ is a term used to refer to a set of cortical areas including the pSTG and parts of the inferior parietal lobe, namely the angular and supramarginal gyri. It receives and integrates information from the visual, auditory, somatosensory systems as well as the thalamus and the limbic system. It is known, for instance, for its role in the processing of emotional facial expressions and theory of mind operations (Roumazeilles et al., 2021). Interestingly, Patel et al. (2019) compared humans’ and macaques’ brains and found that the pSTS-TPJ region has expanded and rearranged along brain evolution. These authors proposed that anatomical and functional reorganization of pSTS-TPJ has served as a basis for the consolidation of a third processing stream that allowed the emergence of increased social abilities in humans. pSTS-TPJ is conceived as a hub that processes facial and other biological motion information allowing us to generate internal models of social scenarios based on previous empirical and multisensory social interactions. Somehow, this area bridges together the information required for understanding the mental states (e.g., intentions, beliefs, desires) of others and thus for the emergence of meaning about our social environment. It is also involved in our ability to imitate, which mainly relies on cross-modal associations of sensorimotor information (e.g., visuomotor associations for facial or gestural imitation and audiomotor associations for vocal imitation) and is crucial to social adaptation and learning (Michon et al., 2022).

FMRI studies in humans have reported that brain activity in response to the integration of people-related (vs. objects-related) information, particularly faces and voices, is restricted to the right pSTS, suggesting a functional lateralization of this area (Landsiedel & Koldewyn, 2023; Watson et al., 2014). A structural asymmetry of the depth of STS was also reported in human newborns, infants, children and adults, this sulcus being deeper in the right than in the left hemisphere (Bodin et al., 2018; Dubois et al., 2010; Leroy et al., 2015). Since they only observed a barely visible asymmetry in macaques, Leroy et al. (2015) argued in favor of the genetic origin of this human-specific STS asymmetry that could have favored the evolution of increasingly sophisticated communicative and social cognition abilities. However, Hopkins et al. (2023) recently reported a small but significant rightward asymmetry in STS depth in a sample of 292 chimpanzees, challenging the idea that this asymmetry pattern is uniquely human. Like humans, chimpanzees also use multimodal signals to communicate. To understand each other, they need to integrate their conspecifics’ facial, vocal, and gestural behaviors. The right STS is known for its fundamental role in interpreting the meaning and communicative intentions of these multimodal stimuli (Redcay, 2008). The presence of this rightward asymmetry in STS depth in chimpanzees (although smaller compared to humans) is therefore more suggestive of gradual and continuous structural changes along nonhuman primate brain evolution. It is noteworthy that nonhuman primates living in larger social groups show an increased volume of white matter (Meguerditchian et al., 2021) and gray matter, especially in the mid-STS and prefrontal regions (Sallet et al., 2011). A deeper understanding of the evolutionary trajectory in the degree of pSTS asymmetry and the relation between its depth and prosocial behaviors in nonhuman primates have the promising potential to shed new light on the origin of our social brains.

#### From the grounding of meaning to semantic networks

Evidence supporting the existence of a third visual pathway for social perception comes from studies of both human and nonhuman primate brains. Interestingly, Pitcher and Ungerleider (2021) raised the possibility of lateralization of the third visual pathway in the right hemisphere, leaving the question of the potential role of this stream in the left hemisphere unanswered. Different populations of neurons within the left pSTS were identified that fired in response to mouth or eye movements. Remarkably, those neurons that fired for mouth (but not for eyes) movements also responded to conspecific voices (Rennig & Beauchamp, 2018, 2022; Zhu & Beauchamp, 2017). Similar multisensory processing of visual and auditory communicative signals has been reported in rhesus macaques (Froesel et al., 2022; Ghazanfar et al., 2005, 2008). As proposed elsewhere (Aboitiz, 2018a, 2018b), due to its privileged projection along the visual system processing dynamic faces and the auditory system processing voices, the third visual stream for social perception in the language-dominant hemisphere represents a well-suited neural network to support audiovisual integration of human speech (Kausel et al., 2024; Michon et al., 2024) and nonhuman primates’ lip-smacking for communicative purposes (Michon et al., 2022).

Here, we propose that in addition to its involvement in multimodal speech perception in our contemporary human brains, the left pSTS has played a critical role in the emergence of meaningful communicative behaviors and the consolidation of a proto-lexicon and primitive semantics in our ancestors’ brains. As a multimodal region at the interface of the ventral auditory and visual pathways, pSTS has strengthened the referring–referred mapping, namely associations between acoustic properties of conspecific vocalizations produced in a particular context (e.g., alarm call) and the visual information about surrounding objects, actions, or events (e.g., the presence of a predator). The medial temporal gyrus (MTG) has progressively appeared and expanded along brain evolution (Roumazeilles et al., 2020) and its strengthening projections toward frontal articulatory regions via the arcuate fasciculus in humans’ left hemisphere have facilitated the transformation of phonological information into speech motor commands (Aboitiz, 2018a; Skeide & Friederici, 2016), allowing the reproduction or imitation of intentional communicative vocalizations within a given social group. Then, learned audio-visual-motor associations between vocalizations and visual representations along the pSTS may have contributed to the apparition of a primitive lexicon providing meaning to phonological and articulatory sequences. With increasingly organized societies, this proto-lexicon became more complex involving multisensory information from our environment and eventually abstract concepts. The expansion of this lexicon may have increased the exposure to conventionalized articulatory sequences consolidated by phonological memory and allowed the use of vocalizations to refer to objects, people, or events even in their absence in the visual scene.

In line with our proposal, Petrides (2023) recently argued in favor of a polysensory neural population in the left STS that integrates highly processed visual information and highly processed auditory information. He claimed that semantic processing requires both sensory-specific information and multisensory integration of information and that our ability for semantics has evolved from the ventral expansion of the polysensory STS to form the adjacent MTG.

## DISCUSSION

In earlier works, we have proposed the hypothesis that in the Homo lineage, the growth of auditory–vocal connectivity via an expansion of the arcuate fasciculus and related tracts of the dorsal auditory pathway enabled our ancestors with an increasing vocal repertoire (Aboitiz, 2018a; Aboitiz & García, 1997; see also Rilling et al., 2008; Scott et al., 2012). In light of this evidence, we suggest that the increasing plasticity of vocal (and manual) behavior in hominins was accompanied by the development of an amplified network in which different sensorimotor components (particularly visuomotor and auditory–vocal) interacted to provide the early meanings that led to early human language, possibly using a combination of gestures and learned vocal calls. In this article, we develop an argument highlighting the co-option of the multisensory STS, the anterior temporal lobe (ATL), and the TPJ to support an interface “translating” linguistic elements like words or proto-words into a multimodal network depicting real objects or events (see also Aboitiz, 2018b).

As mentioned, several previous reports have highlighted the role of the STS and the ATL in multimodal integration, working as an interface between the ventral auditory stream in the STG and the ventral visual stream along the inferior temporal gyrus (see Binder & Desai, 2011; Binder et al., 2009; Perrodin et al., 2015; Petrides, 2014). Compared to nonhuman primate, the human temporal lobe has expanded, yielding the adjacent MTG (Roumazeilles et al., 2020; Sierpowska et al., 2022), which represents an amplification of this multisensory region. In this line, some authors have proposed that in the left hemisphere, this region became critical for establishing associative links between auditory inputs and visual stimuli, which may have been critical for social communication in early humans (Petrides, 2014, 2023). Together with gestural communication (Becker & Meguerditchian, 2022; Meguerditchian, 2022), the STS and adjacent regions may have provided a primitive scaffolding linking vocalizations or gestures with visual and other modalities. (Recall that neurons in the macaque STS may respond to auditory, visual, somesthetic, or a combination of these stimuli; Bruce et al., 1981.) Consistent with this notion, in nonhuman primates, complex visual and social input to the STS works by enhancing or inhibiting the production of vocal calls, which may represent an early scaffolding for reference signals, such as alarm calls depicting specific events (Fischer & Price, 2017; Froesel et al., 2022). An interesting instance is that of vervet monkeys, which have different calls depicting specific predators. While the structure of the calls is innate, their predator references are learned through social experience (Seyfarth et al., 1980). It remains to be determined whether the STS mediates the development of such referential communication as we would suggest.

In humans, there is considerable evidence that the semantic network partly relies on the activation of the STS, ATL, and MTG (Binder et al., 2009; Bryant & Preuss, 2018; Hodgson et al., 2023; Lambon Ralph, 2014; Lambon Ralph et al., 2017; for a complementary review on the evolution of STS and MTG for semantic, see Petrides, 2023). Furthermore, the ventral language stream is known to project to the pars triangularis (anterior Broca’s area), receiving auditory and visual input via the extreme capsule, which participates in selecting lexico-semantic information in the human left hemisphere (Kostopoulos & Petrides, 2016). Rauschecker and Scott (2009) suggested that the sensorimotor role of the action stream in the visual system could be extended to the dorsal stream of the auditory cortex performing “auditory-motor transformations in verbal working memory tasks that involve articulatory representations” (p. 721). They argued that these transformations may be based on a multisensory reference frame. Therefore, we consider that in human brain evolution, the STS and neighboring sensorimotor and social brain areas were pivotal for the development of word-related concepts depicting objects, events, or names, which eventually expanded the semantic network into widespread representations across the cerebral cortex (Shahdloo et al., 2022; Small et al., 1995). It was recently reported that STS exhibits effective connectivity to regions of the inferior parietal lobe such as areas TPOJ1, STV, PSL and PGi (depicted in Figure 2), “which are language-related regions involved in semantic representations about objects, faces, and so on using multimodal information, and which then connect to Broca’s area, especially to area 45” (Rauschecker & Afsahi, 2023, p. 1888). Interestingly, STS also shows effective connectivity to areas TGv and TGd in the ATL (Rolls et al., 2023).

An influential theory in the field of semantic cognition recognizes the ATL as a modality invariant hub performing transmodal computations that are meant to control the use of semantic multisensory knowledge in a context-dependent manner (Lambon Ralph, 2014; Lambon Ralph et al., 2017). As mentioned earlier, both visual and auditory ventral streams seem to be organized along a posteroanterior gradient with increasingly complex processing of sensory inputs in anterior parts of the temporal lobe. According to these anatomo-functional schemes, supported by previous evidence reviewed in this perspective article, we do not exclude the possibility of a similar organization for the semantic network. A posteroanterior gradient may exist in the human temporal lobe, from low-level sensorimotor integration of multimodal social signals (e.g., not only biological motion of bodies and faces but also voice processing) in posterior regions of the STS and MTG to higher-order, transmodal semantic control for time and context-appropriate use of multimodal experiential knowledge in anterior portions of the temporal lobe, especially ATL. For instance, if you order “some coffee” at a restaurant, it is very unlikely that the waiter will bring you a cup of coffee beans (which would be semantically correct but pragmatically clumsy). However, suppose you are a barista opening the coffee machine and you ask your colleague for some coffee. Hopefully, in that case, he will bring you some coffee beans to refill the machine bean container. According to the transmodal hub hypothesis, the ATL would supervise the pragmatic decision that better suits the context or task.

Despite promising progress in addressing some knowledge gaps, research on multimodal primate communication remains relatively uncommon. Some authors believe that theories of language evolution are unlikely to advance unless the field of primate communication research recognizes and addresses these knowledge gaps (Liebal et al., 2022). Theories suggesting that human language may have a multimodal origin are beginning to receive more support (Fröhlich et al., 2019; Wacewicz & Zywiczynski, 2017). This could introduce a theoretical shift and potentially promote the development of methodological approaches to empirically assess the multimodality of primate communication. In this perspective, we propose a new research agenda, focusing on the lateralization of a third lateral pathway running along the STS and TPJ, particularly concerning its functions in the language-dominant left hemisphere. Furthermore, comparative studies about this region may provide important insights into the evolutionary origin of our most cherished capacity, which is human language.

## FUNDING INFORMATION

Francisco Aboitiz, Agencia Nacional de Investigación y Desarrollo (https://dx.doi.org/10.13039/501100020884), Award ID: Fondecyt Regular 1210659. Maëva Michon, Agencia Nacional de Investigación y Desarrollo (https://dx.doi.org/10.13039/501100020884), Award ID: Fondecyt Postdoctoral 3201057.

## AUTHOR CONTRIBUTIONS

**Francisco Aboitiz**: Conceptualization: Equal; Funding acquisition: Equal; Investigation: Equal; Writing – original draft: Equal; Writing – review & editing: Equal. **Maëva Michon**: Conceptualization: Equal; Funding acquisition: Equal; Investigation: Equal; Writing – original draft: Equal; Writing – review & editing: Equal.

## TECHNICAL TERMS

Multimodal: Requiring the involvement of multiple sensory modalities.
Reenactment: Bringing past experiential knowledge to the present.
Active inference: Learning by minimizing the difference between predictions and sensory inputs through actions and belief updates.
Transmodal: Modality independent.
